# Biomarkers for the Clinical Diagnosis of Alzheimer’s Disease: Metabolomics Analysis of Brain Tissue and Blood

**DOI:** 10.3389/fphar.2021.700587

**Published:** 2021-07-21

**Authors:** Yang-Yang Wang, Yan-Ping Sun, Yu-Meng Luo, Dong-Hui Peng, Xiao Li, Bing-You Yang, Qiu-Hong Wang, Hai-Xue Kuang

**Affiliations:** ^1^ Key Laboratory of Chinese Materia Medica Ministry of Education, Heilongjiang University of Chinese Medicine, Harbin, China; ^2^ School of Traditional Chinese Medicine, Guangdong Pharmaceutical University, Guangzhou, China

**Keywords:** alzheimer’s disease, candidate diagnosis biomarker, metabolomics, brain and blood samples, clinical screening

## Abstract

With an increase in aging populations worldwide, age-related diseases such as Alzheimer’s disease (AD) have become a global concern. At present, a cure for neurodegenerative disease is lacking. There is an urgent need for a biomarker that can facilitate the diagnosis, classification, prognosis, and treatment response of AD. The recent emergence of highly sensitive mass-spectrometry platforms and high-throughput technology can be employed to discover and catalog vast datasets of small metabolites, which respond to changed status in the body. Metabolomics analysis provides hope for a better understanding of AD as well as the subsequent identification and analysis of metabolites. Here, we review the state-of-the-art emerging candidate biomarkers for AD.

## Introduction

**FIGURE 1:**
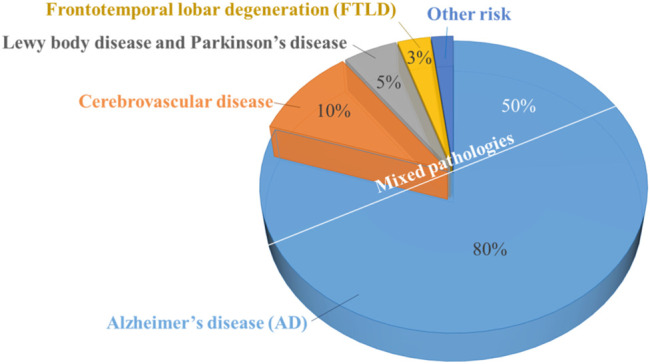
Common disease causes of dementia percentage.

Alzheimer’s disease (AD) is a progressive neurodegenerative disorder. It is the most common form of dementia (comprises about 60–80% of cases) (Figure 1). AD is characterized by difficulties in memory recall, language, thinking, and other problem-solving abilities that severely affect a person’s ability to perform daily activities (Alzheimer’s Association, 2020). This is a major public health problem that causes devastating physical and economic consequences for patients, their families, and society.

**FIGURE 2:**
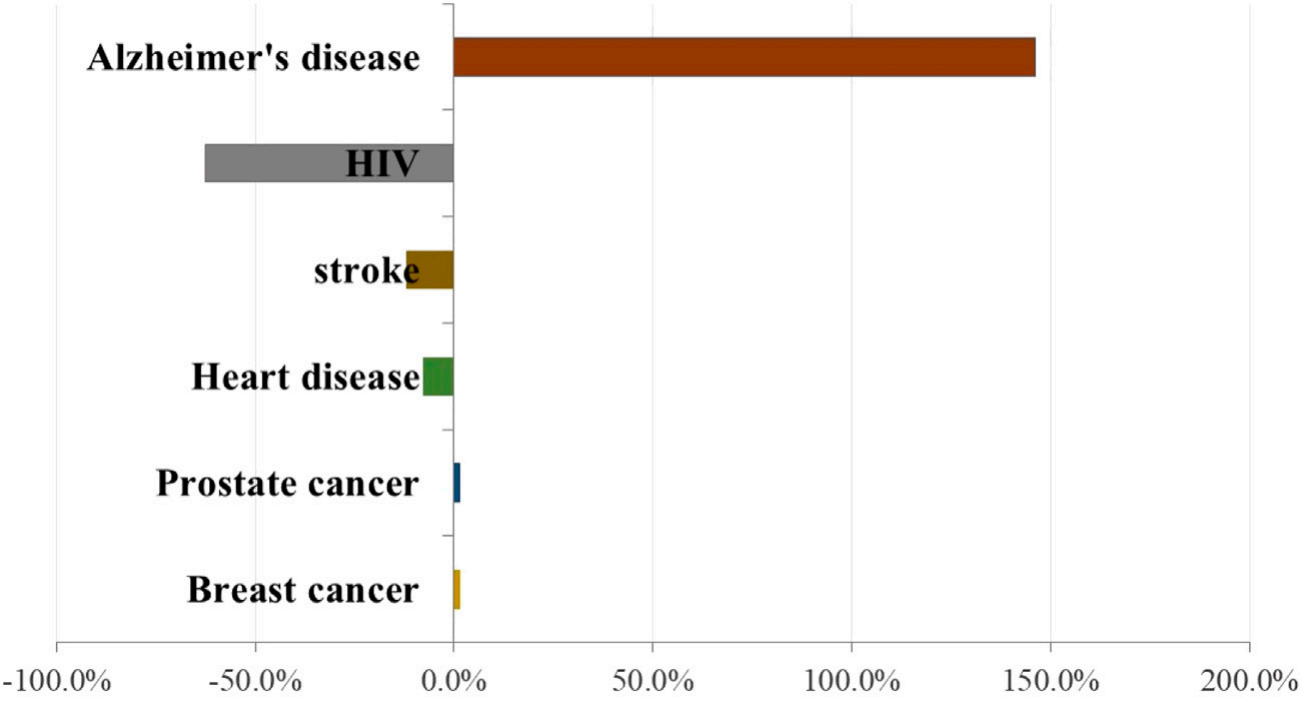
Percentage change in reported deaths related to specific diseases between 2000 and 2018. Based on Alzheimer’s disease reporter association in 2020 (Alzheimer’s Association, 2020).

The total estimated cost of dementia care worldwide was ∼$1 trillion in 2018, and this cost is expected to double by 2030 (Peña-Bautista et al., 2020). The World Health Organization estimates ∼50 million people worldwide have dementia and that ∼150 million people will have dementia by 2050 (Peña-Bautista et al., 2020). According to the Alzheimer’s Association, although deaths from other major diseases (e.g., heart disease, cancer, stroke, and infection by the human immunodeficiency virus) have declined significantly or remained approximately identical in the past decade, the number of deaths caused by AD increased by 146% between 2000 and 2018 (Figure 2). This increased prevalence of mortality from AD is due, in large part, to AD becoming a common cause of death among the aging population as well as greater accuracy in diagnosing clinical dementia and recording the cause of demise (Stamate et al., 2019).

AD is thought to begin ≥20 years before symptom onset and become worse with time (Villemagne et al., 2013; Gordon et al., 2018). The prodromal phase of AD would provide a critical “window of opportunity” for therapeutic intervention to delay AD onset and slow the progress of neurodegeneration (Sperling et al., 2011; Sevigny et al., 2016). Therefore, it is essential to explore more effective preclinical-stage diagnostic methods, which can be used to slow down or prevent dementia onset.

## Stages of Alzheimer’s Disease

**FIGURE 3:**
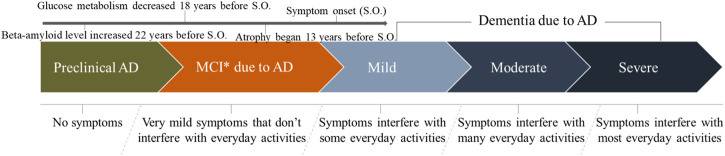
The development progression of Alzheimer’s disease in clinical research.

With respect to AD progression, three broad stages have been outlined in the new guidelines for the diagnosis (Albert et al., 2011; Jack et al., 2011; McKhann et al., 2011) (Figure 3). Phase 1 is preclinical AD (no symptoms and brain change is unnoticeable to the person affected but he/she carries a high risk of developing AD). Phase 2 is mild cognitive impairment (MCI) due to AD (changes in memory and thinking abilities are very mild and do not interfere with daily activities). Phase 3 is dementia due to AD (changes in memory, thinking, and behavioral abilities are clear in various degrees and interfere with daily activities). In addition, the dementia phase can be broken down further into “mild,” “moderate,” and “severe” stages, which reflect the degree to which symptoms interfere with the ability to undertake everyday activities.

With regard to the preclinical phase of AD, a recent study involving rare genetic mutations found that amyloid beta (Aβ) levels were increased significantly, starting 22 years before symptoms were expected to develop (Gordon et al., 2018). Moreover, individuals with these genetic mutations usually develop symptoms at the same (or nearly the same) age as their parent who suffered AD. Meanwhile, glucose metabolism decreases and brain atrophy begins 18 and 13 years before expected symptom onset, respectively (Gordon et al., 2018). Data have shown that the rate of conversion from MCI to AD occurs at 10–15% per year, with 80% conversion by the sixth year of follow-up, approximately (DeCarli, 2003). Therefore, the early diagnosis and identification of AD patients requires careful screening.

In this review, we update the state of diagnostic methods (initial assessment, screening tools, and brain imaging) and biomarker development (based on brain tissue and blood) for preclinical AD. In addition, we make the case for consistency in collection of clinical data for all types of AD biomarkers to allow more thorough and rapid comparisons between studies.

## Metabolomics Analysis and Alzheimer’s Disease

The US Food and Drug Administration defines a “biological marker” as an indicator of normal biologic or pathogenic processes or pharmacological responses to a therapeutic intervention. In general, biomarkers in the body are essential not only for the early diagnosis of a disease but also in the assessment of prognosis, classification of disease progression, and disease-modifying treatment (Cummings, 2011; Zhang et al., 2012). Several biochemical processes, such as the metabolism of amyloid precursor protein (APP), phosphorylation of tau protein (p-tau), oxidative stress, impaired energetics, mitochondrial dysfunction, inflammation, dysregulation of membrane lipids, and disruption of neurotransmitter pathways, are affected in AD (de la Monte and Tong, 2014). These changes can be reflected by biomarkers. Therefore, the metabolomics analysis (MA) provides a new method to reveal the nature of a multifactorial disease.

MA is an emerging high-throughput “omics” technology. It is used to comprehensively and simultaneously identify metabolites within a biological system that show altered expression from the genomic, transcriptomic, and proteomic system in a high-throughput pattern (Fiehn, 2002; Beckonert et al., 2007). Recently, MA has been shown to aid discovery of novel biomarkers and explore the potential mechanism of disease by analyzing the entire biological system rather than single metabolites (Snyder et al., 2014). In addition, MA provides detailed biochemical information about drug candidates, therapeutic effects, and side effects during the discovery and development of drugs (Kaddurah-Daouk and Krishnan, 2009), and offers insights into the full complexity of the disease phenotype (Eckhart et al., 2012).

Several MA studies have reported that AD involves disordered metabolism of branched-chain amino acids (Tynkkynen et al., 2018), phosphatidylcholine (Mapstone et al., 2014), glycerophospholipids, and sphingolipids (Orešič et al., 2011; Varma et al., 2018). MA is a new “frontier” in the diagnosis of multifactorial chronic disease because hundreds of metabolites can be detected rapidly and simultaneously (Hassan-Smith et al., 2012).

## Assessment of Alzheimer’s Disease in Clinical Practice

### Cognition Assessment in Alzheimer’s Disease

The initial diagnosis of AD commonly involves family, close friends, or the general practitioner of a person who is concerned about memory and behavioral changes in daily life. Several diseases featuring delirium, depression, and pain syndromes can present with the similar impairment in memory, perception, and cognitive skills observed in AD. In the early phase of AD, changes in memory (particularly short-term memory) and in behavior and mood (confusion regarding the time of day/familiar places, aphasia, forgotten items, and storing items in inappropriate places) can occur. Then, with aging, these symptoms worsen.

AD can be excluded rapidly in older patients by urine tests, blood tests, and physical examination. In addition, a brief cognitive test may also be conducted, for example, the Mini-Mental State Examination (MMSE) (Andrade-Moraes et al., 2013), to screen for the presence and level of cognitive impairment. Once other likely causes have been excluded, patients are referred to a geriatrician or neurologist for further testing. These specialists will undertake a lengthy clinical test and may require a neuropsychological assessment, which concentrates on the specific domain of cognition. Behavioral and functional assessments may also be implemented (though these are used more commonly in more severe stages of dementia). Behavioral assessment involves investigation of the noncognitive part of dementia (including personality, emotion, and psychotic symptoms) and unusual behaviors as well as obstacles to sleeping, eating, and sexual activity (Fuller et al., 2019). Assessment of noncognitive characteristics has improved the diagnostic accuracy of AD as well as aiding assessment of care requirements and distinguishing between different causes of dementia. The usual assessment approaches and applications for AD in clinical practice are shown in Table 1.

**TABLE 1:** The usual assessment approaches of cognition domain and application in clinical.

Screening methods	Application in clinical	Advantage	Disadvantage	Difference
Mini-Mental State Examination (MMSE)	Differentiate from Lewy bodies (DLB) Ala et al. (2002), Palmqvist et al. (2009), vascular dementia, or dementia due to Parkinson’s disease (PD) Jefferson et al. (2002)	Convenient (approximate 10 min)	Affected by education level, age practice effect, and ceiling effect (false-negative rates)	—
Montreal Cognitive Assessment (MoCA)	Measure for MCI and cognitive impairment	1) Convenient (approximate 10 min); 2) More utility and accuracy	1) The cut-off score of 26 is too high and may increase the potential for false-positive diagnosis Rossetti et al. (2011). 2) Affected by education, age, social, and ethno-racial	Compared with MMSE, it’s had the more comprehensive assessment of cognition, a lower ceiling effect, the ability to detect MCI.
Clinical Dementia Rating (CDR)	Assess stage of dementia syndrome	Semi-structured global rating measures for the diagnosis and for determining the severity of dementia Hughes et al. (1982)	—	—
Clock Drawing	Combine with MMSE in dementia screening tests	1) A short test; 2) with minimal training	Some extent subjectivity	As part of the 7-min screen and Mini-Cog test and MoCA.
Seven-Minute Screen	A quick screening tool is often used in primary care to determine the presence of dementia Meulen et al. (2004)	1) A short test; 2) with minimal training	—	—
Alzheimer’s Disease Assessment Scale (ADAS-Cog)	Assess AD severity (staging) degree and the primary outcome in clinical trials of anti-dementia drugs	More Professional	1) Time-consuming (approximately 30 min) and prone to practice effect; 2) Not a substitute for extensive neuropsychological testing Peña-Casanova (1997)	1) The test requires greater training to administer and score than MMSE; 2) The test is more thorough than the MMSE
Psychogeriatric Assessment Scales (PAS)	Assessments of the clinical changes seen in dementia	With minimal training	1) Patients need to be fluent in English and need as an objective informant as possible; 2) May be influenced by premorbid intelligence and level of education	—
Dementia Rating Scale (DRS)	Detect MCI in subjects Matteau et al. (2011)	Increased discrimination and sensitivity to differentiate individuals with substantial cognitive deficits	—	—
Mini-Cog	—	1) Brief test; 2) Takes less time (less than 5 min)	No useful for writing or drawing difficult individual	1) It takes less time than MMSE Borson et al. (2003); 2) less susceptible to language problems and educational level than the MMSE.
Rowland Universal Dementia Assessment Scale (RUDAS)	More widely applicable assessment methods	1) Easily administered Storey et al. (2004); 2) Valid test people from culturally and linguistically diverse populations (Naqvi et al., 2015)	—	Overcome the cultural, educational level, and language problems sometimes encountered in other tests such as the MMSE
Neuropsychological Battery (NB) and Other Tests of CERAD	1) Discriminate between healthy controls, mild and moderate dementia Moms et al. (1989); 2) Distinguishing early AD from normal elderly	1) Quite comprehensive; 2) Language-free (such as English, Chinese, Japanese, Italian, and German)	Time-consuming	More accurate than the MMSE in discriminating progressing MCI subjects from controls Paajanen et al. (2014)

Also, imaging of the brain can be carried out by computed tomography (CT), electroencephalography (EEG), magnetic resonance imaging (MRI), and single-photon emission computed tomography (SPECT). However, these imaging modalities are expensive, time-consuming, and may be available only in major cities as part of research programs.

### Diagnostic Biomarkers in Alzheimer’s Disease

Several diagnostic methods for AD are available based on the measurement of the Aβ level in cerebrospinal fluid (CSF) (Hulstaert et al., 1999) and “neurofibrillary tangles” (Caselli et al., 2017), which begin to form years before some symptoms of dementia appear (Perrin et al., 2009). MRI can be used to quantify metabolic abnormalities for measurement of brain atrophy (Reiman and Jagust, 2012). SPECT (Román and Pascual, 2012) and positron emission tomography can be employed to measure the rate of glucose metabolism and Aβ burden (with the radiotracers Pittsburgh compound B and 18F-Florbetapir) (Bruno et al., 2010). Unfortunately, those diagnostic methods are limited because they are invasive, time-consuming, and expensive.

Based on the “amyloid hypothesis,” the Aβ-42 isoform of Aβ has been focused upon as a biomarker for measurement (Hardy and Selkoe, 2002). In CSF, a low Aβ-42 concentration as well as increased total tau (t-tau) and p-tau levels could indicate conversion from MCI to AD (Blennow et al., 2001; Shaw et al., 2009). Recent studies have reported that an increased t-tau level in CSF is observed in neuronal degeneration, but Aβ deposition is detected in normal ageing (Aizenstein et al., 2008). However, Aβ-42 in CSF is not specific to AD because it can be observed in neuronal degeneration and normal ageing. The accuracy of AD diagnosis can be increased ≤90% (in tandem with increasing costs to patients) if analyses of specific protein levels are combined with brain imaging (Cedazo-Minguez and Winblad, 2010). AD can be diagnosed clearly only after death through examination of brain tissue and pathology in an autopsy. Hence, even though use of blood-based biomarkers is attractive, they cannot be used to detect preclinical AD with the requisite sensitivity and specificity (Thambisetty and Lovestone, 2010).

### Neuropathologic Evaluation of Alzheimer’s Disease

Accumulation of protein fragments of Aβ (“Aβ plaques”) outside neurons and accumulation of an abnormal form of tau protein (“tau tangles”) within neurons are two of several brain changes associated with AD. Aβ plaques are considered a hallmark feature in the brain of AD patients. They consist mainly of aggregated Aβ peptides of size 4 kDa (ranging from 39 to 43 amino acids). Aβ peptides are the normal breakdown products of APP. The latter is a widely expressed transmembrane protein and cleaved by α-, β-, and γ-secretase. APP is cleaved within the Aβ domain by α-secretase to release the neurotrophic ectodomain of APP into the CSF and prevent Aβ generation (Sisodia, 1992). In addition, β- and γ-secretase act with APP to generate the N and C termini of the Aβ peptide (Haass and Selkoe, 1993). The specific site of cleavage by γ-secretase is variable and generates mostly Aβ-40 peptide (Mann et al., 1996). Approximately 10% of secreted Aβ is Aβ-42, which aggregates readily and acts as a nidus for plaque formation by recruiting Aβ-40 (Jarrett and Lansbury, 1993; Iwatsubo et al., 1994). However, the longer forms of Aβ1–42 are produced in excess and aggregate into oligomers and then form amyloid fibrils in AD (Blennow and Hampel, 2003). Plaque deposition usually begins in the isocortex (frontal, temporal, and occipital lobes of the gray matter of the cortex) and then, in the entorhinal cortex, hippocampal formation, amygdala, insular, and cingulated cortices can be detected (Serrano-Pozo et al., 2011).

Tau tangles (also called neurofibrillary tangles) are swirls or strands of fibers within neurons. They consist mainly of aggregates of microtubule-associated tau protein. The severity of AD symptoms is closely related to the extent of deposition of tau tangles (Arriagada et al., 1992; Serrano-Pozo et al., 2011). In AD, tau protein appears to be abnormally hyperphosphorylated, with phosphate groups attached at specific sites on the protein. Aβ plaques and tau tangles induce damage and death of neurons by interfering with neuron-to-neuron communication and blocking the transport of nutrients and other essential molecules within neurons, respectively (Hyman and Trojanowski, 1997).

It has been proposed that Aβ may begin to accumulate before accumulation of abnormal tau protein and that increasing Aβ accumulation is related to subsequent increases in tau protein (Sato et al., 2018; Hanseeuw et al., 2019). In addition, microglial infiltration, widespread loss of synapses and neurons, and brain shrinkage occur. The toxicity of Aβ and tau proteins can activate microglia (which are the immune system cells in the brain). The main function of microglia is to clear toxic proteins and debris from dead and dying cells. Chronic inflammation may occur if microglia cannot maintain this function. However, shrinkage or atrophy of the brain may develop if synapses and neurons are lost, and normal brain function may be damaged further by the reduced metabolism of glucose (the main fuel in the brain).

## Biomarker-Based Metabolomics Analysis

### Brain and Blood Samples in Alzheimer’s Disease Studies

Metabolites are intermediates and products of metabolism required for cell growth and are the basis of many other biological components (Mamas et al., 2011). Due to their close relationship to the host’s phenotype, the profile of metabolites shows the current physiological state of a cell and the final result of upstream biological information, which flows from the genome to the transcriptome, proteome, and metabolome (Brink-Jensen et al., 2013). Therefore, metabolites are a relatively more suitable target for phenotype-based research than transcription factors and proteins, both of which are information messengers and executors of biochemical reactions (Enche Ady et al., 2017). An imbalance of metabolic homeostasis is a precursor for disease, so assessment of changes in metabolomes through MA may become a major application for disease description and development of novel therapeutic strategies (Kiehntopf et al., 2013).

A preclinical biomarker is essential for the early diagnosis, stratification, and prevention of AD. The biomarkers for preclinical studies are levels of tau protein and Aβ, as well as structural and functional MRI (Franceschi et al., 2000). The predictive value of these biochemical and imaging markers is very limited because they reflect the final stage of AD (Winblad and Lönneborg, 2014). Hampel and colleagues showed that the concentration of Aβ1–42, t-tau and p-tau in CSF could be used to differentiate people with MCI or AD from healthy older individuals with 80% sensitivity (Hampel et al., 2008). These biomarkers cannot be applied in an early diagnosis of AD because they are detected only when brain functionality is compromised irreversibly (Contino et al., 2013).

CSF and plasma contain the richest source of biomarkers. CSF is an ideal source of various biomarkers for events occurring in AD because it: 1) communicates directly with superficial portions of the brain; 2) it does not pass through the tightly regulated blood–brain barrier (BBB); 3) reflects the biochemical changes that occur in the brain (Aluise et al., 2008; Blennow et al., 2010). However, CSF collection is invasive because it involves a lumbar puncture, which requires patient compliance and can be a challenge in older people with arthritic spines (Henriksen et al., 2014) as well as risking the complications of headaches and meningitis (de Almeida et al., 2011). In addition, patients with low CSF pressure/volume can have a high failure rate of CSF collection (≥20%) and a higher risk of sequelae (Hansson et al., 2006). Besides, CSF collection requires significant clinical skills and use of a sterile method to minimize the risk of headaches, meningitis, epidural abscess, subdural hematoma, and death (Kuntz et al., 1992; Pandian et al., 2004; Martín-Millán et al., 2005). Therefore, biomarker-based brain research and clinical applications for the early diagnosis of AD are limited.

Identification of relevant biomarkers in blood samples has attracted attention in AD research because collection of blood samples is noninvasive, inexpensive, rapid, and influenced less by external factors. Besides, a blood sample is a stable source for repeat measurements. Nevertheless, there is evidence that BBB function is disrupted with aging and that the degree of cognitive impairment results, ultimately, in increased permeability (Baird et al., 2015). This observation reveals the close relationship between the brain and blood. Approximately 500 ml of CSF is absorbed into blood each day, and the small-sized metabolites (e.g., peptides) present in the brain can be detected in plasma or serum (Zipser et al., 2007; Voyle et al., 2016).

### Biomarkers Based in Brain Samples in Clinical Research

Lipids have key roles in maintaining normal physiological functions in the body, such as energy storage, signal transduction, maintenance of cell membrane structure, and cell transport (Brügger, 2014). Disordered lipid metabolism is closely related to neurological diseases (e.g., AD and Parkinson’s disease) and affects cognitive function (Gaamouch et al., 2016). Lipid metabolites and pathways strategy (LIPID MAPS; www.lipidmaps.og/) can be used to study the role of lipids. In this way, lipids can be classified into eight main categories: fatty acids, glycerolipids, glycerophospholipids, sphingolipids, saccharolipids, polyketides, prenol lipids, and sterols (Fahy et al., 2009). Glycerophospholipids, sphingolipids, and cholesterol are localized mainly to neuronal membranes and myelin.

Recent studies have indicated that disruption of the metabolism of cholesterol and lipids in the brain is closely related to the generation, deposition, and clearance of Aβ and, finally, leads to neuronal dysfunction (Wellington, 2004; Han, 2010; Song et al., 2012; Wong et al., 2017). A series of studies has claimed that APP processing may occur in cholesterol-rich regions known as “lipid rafts.” Intracellular cholesterol may modulate APP processing directly by regulating secretase activity or affecting the trafficking of secretase or APP (Francis et al., 2002; Edbauer et al., 2003; Li et al., 2003; Cordy et al., 2006). Studies have suggested that intracellular cholesterol regulates the metabolism of Aβ peptides by shifting APP from α- to β-cleavage products, which accumulate as amyloid plaques in the brains of AD patients (Panza et al., 2006). Statins (inhibitors of cholesterol biosynthesis) can reduce the morbidity of AD by ≤70% (Jick et al., 2000; Wolozin et al., 2000). There is strong evidence that AD pathogenesis implicates a genetic variant of apolipoprotein E (ApoE). Apo4 (encoded by the Apoε4 allele) is the highest genetic risk factor for late-onset AD (Corder et al., 1993). In the brain, ApoE is the main cholesterol-carrier protein, which promotes the transfer of cholesterol from astrocytes to neurons. ApoE can also bind Aβ in a genotype-dependent manner. ApoE3 has a higher affinity and greater ability to clear Aβ than that of ApoE4 (Castellano et al., 2011). ApoE2 has a protective effect against AD (Conejero-Goldberg et al., 2014).

Varma and colleagues undertook a study on quantitation of targeted metabolomics in brain tissue using an AbsoluteIDQ® p180 kit (Biocrates Life Sciences). The study cohort was 15 AD patients, 14 healthy controls (HCs), and 15 individuals with asymptomatic Alzheimer’s disease. Two main classes of sphingolipids and glycerophospholipids with 26 metabolites were documented, including sphingomyelin (SM) with acyl residue sum C16:0, C18:1, and C16:1 and hydroxy-sphingomyelin with acyl residue sum C14:1 (SM (OH) C14:1), which could be used to discriminate AD patients and HCs with 83.33% accuracy. Sphingolipids were implicated in biologically relevant pathways in AD: p-tau, Aβ metabolism, calcium homeostasis, acetylcholine biosynthesis, and apoptosis (Varma et al., 2018). Toledo and colleagues reported that increase in levels of SM C16:0 and SM (OH) C14:1 in the temporal cortex of AD patients was related to brain atrophy, cognitive decline, and conversion from MCI to AD (Toledo et al., 2017). Another study of SMs in the brain tissue of AD patients found that carrying ApoE ε4 alleles could lead to increase in levels of ceramide (C22:0) and sulfatide but lower ceramide (C24:0) levels. ApoE ε4 carriers may be important when investigating lipid levels in CSF (Bandaru et al., 2009). On an average of 4.5-year follow-up, longitudinal cohorts of aging and dementia studies found that higher baseline of 3 glycerophospholipids [PC aa C30:0, PC ae C34:0, and PC ae C36:1] and 1 acylcarnitine (C14:2) was present in both the postmortem brain and antemortem blood, which could predict a lower risk for AD. However, only C14:2 was associated with protection against AD, and three glycerophospholipids showed opposite results between brain tissue and blood (Huo et al., 2020). Kaddurah-Daouk and colleagues studied metabolomic changes in autopsy-confirmed AD with 15 AD patients and 15 people not suffering from dementia. Analyses of CSF samples suggested that the level of norepinephrine (NE) and dopamine pathway–related metabolites was reduced significantly in AD cases. In addition, reduced levels of tryptophan, NE, and indoleacetic acid in the CSF of AD cases could be used to distinguish between different groups with 90% accuracy (Kaddurah-Daouk et al., 2011). However, whether a disorder in the NE pathway is unique to AD needs clarification. Some of those studies are shown in Table 2.

**TABLE 2:** Metabolic biomarker in blood for Alzheimer’s disease patients in clinical.

Metabolites	Subject	Population (sample size)	Sample type	Platform	Implication	Study
Alpha-d-galactosyl undecaprenyl diphosphate, lysoPC (18:1), lysoPC (P-18:0), lysoPC (P-18:0), lysoPE (0:0/22:1(13Z)), CL(8:0/14:0/18:2(9Z,11Z)/18:2(9Z,11Z)	AD, CON	CON = 39; AD = 39	Plasma	UPLC-QTOF/MS	Six identified metabolites could discriminate between patients with different ApoE4 genotypes (4-carriers and non—4-carriers)	Peña-Bautista et al. (2020)
Choline, L-carnitine, S-carnitinium, 2,3,4-trihydroxy-5-(3,4,5-trihydroxybenzoyloxy)benzoic, 5-amino-4-hydroxy-3-(phenylazo)-2,7-naphthalenedisulfonic acid, 4-deoxyphysalolactone, bargustanine, alpha-tocopherol succinate, dilauryl 3,3′-thiodipropionate, rescinnamine, chlorohydrin, brassinin, monomethyl phenylphosphonate, cysteinyl-aspartate, aspartyl-cysteine, nicotinamide ribotide, beta-nicotinamide D-ribonucleotide, lysoPE(20:5/0:0), inositol 1,3,4,5-tetraphosphate, cyasterone, lysoPE(20:0/0:0)	AD, MCI, CON	MCI-AD = 29; CON = 29	Plasma	UPLC-QTOF/MS	Choline was identified as a promising AD diagnosis metabolite; the Holinergic system, energy metabolism, and aminoacids and lipids pathways may be involved in early Alzheimer's disease development	Peña-Bautista et al. (2019)
PCs, PC aa C36:6, PC aa C38:0, PC aa C38:6, PC aa C40:1, PC aaC40:2, PC aa C40:6, PC acyl-alkyl (ae) C40:6, lysophophatidylcholine (lysoPC a C18:2), acylcarnitines (ACs) propionyl AC (C3) and C16:1-OH	AD, MCI, CON	AD = 42; MCI = 74; CON = 66	Plasma	SID-MRM-MS	A set of ten lipids from peripheral blood that predicted phenoconversion to either amnestic mild cognitive impairment or Alzheimer’s disease within a 2–3 year time frame with over 90% accuracy	Mapstone et al. (2014)
The levels of choline, creatinine, asymmetric dimethyl-arginine, homocysteine-cysteine disulfide, phenylalanyl-phenylalanine, and different medium-chain acylcarnitines significant increase; asparagine, methionine, histidine, carnitine, acetyl-spermidine, and C5-carnitine were reduced in serum samples of AD.	AD, MCI, CON	AD = 42; MCI = 14; CON = 37	Serum	CE-MS	It was possible to classify patients according to the disease stage and then identify potential markers	González-Domínguez et al. (2014)
C3, C5, C5-DC, arginine, acylcarnitines, phenylalanine, creatinine, symmetric, imethylarginine (SDMA), phosphatidylcholine ae C38:2	AD, MCI, CON	AD = 15; MCI = 10; CON = 10	Plasma	UPLC - TQ-S/MS	The plasma levels of arginine and valeryl carnitine, age is promising as biomarkers for the diagnosis of AD in older adults, accuracy of 85%	Lin et al. (2019)
GC-MS detected 85 metabolites, whereas UHPLC-MS detected 238 metabolites. AUC = 0.998	AD, MCI, CON	AD = 57; MCI = 28; CON = 57	Plasma	GC-MS and UHPLC-MS	Arachidonic acid, N,N-dimethylglycine, thymine, glutamine, glutamic acid, and cytidine could discriminate AD and CON; thymine, arachidonic acid, 2-aminoadipic acid, N,N-dimethylglycine, and 5,8-tetradecadienoic acid could discriminate MCI and CON	Wang et al. (2014)
All 31 kinds of endogenous metabolites were identified	AD, MCI, CON	AD = 30; MCI = 32; CON = 40	Serum	GC-MS	Hydracrylic acid, 1,4-butanediamine, n-octanoicacid, L-phenylalanine, ribitol, L-ornithine, D-glucose, D-turanose, hexadecanoic acid, propylester, androstenediol, cholesterol successfully distinguished between AD and CON; L-alanine, n-octanoic acid, L-phenylalanine, ribitol, L-ornithine, citric acid, D-glucose, inositol, hexadecanoic acid, propylester, androstenediol distinguished between AD and CON.	Sun et al. (2020)
Long-chain cholesteryl esters (ChEs): ChE 32:0, ChE 34:0, ChE 34:6, ChE 32:4, ChE 33:6, ChE 40:4, et al.	AD, MCI, CON	AD = 35; MCI = 48; CON = 40	Plasma	UPLC- QTOF/MS	A combination of 10 metabolites could discriminate AD patients from controls with 79.2% accuracy	McCrimmon et al. (2012)
C12-DC, C12, PC aa C26:0, acylcarnitines, lysophospholipids, sphingomyelins	AD, MCI, CON	AD = 34; MCI = 20; CON = 25	Plasma	Absolute IDQ p 180 assays	A combination of C12-DC, C12, and PCaaC26:0 could differentiate AD and MCI, with a predictive value of 79%	Costa et al. (2020)
Ceramide C22:0 and C24:0	AD, MCI, CON	AD = 21; AMCI = 17; CON = 25	Plasma	LC/MS/MS	Higher baseline ceramide C22:0 and C24:0 levels were predictive of cognitive decline and hippocampal volume loss	Mielke et al. (2010)

The biomarker studies described above suggest that AD research using brain tissue or CSF has made considerable progress. However, obtaining samples is invasive and carries risks for older patients. Even though such samples may offer direct information on brain status, such samples are unlikely to be used for the clinical diagnosis.

### Biomarkers Based on Blood Samples in Clinical Research

Use of blood-based biomarkers has garnered increasing attention because blood collection is noninvasive, rapid, and carries little risk compared with that based on CSF collection (Humpel, 2011). Blood is a valid source for repeat measurements, thereby making blood-derived biomarkers for AD is highly sought after (Enche Ady et al., 2017). As stated above, the BBB is disrupted with aging and cognitive impairment in AD development, which results in increased permeability and strengthens the communication between blood and the brain (Baird et al., 2015). CSF is absorbed into the blood circulation each day and small-sized peptides (or even proteins) can be detected in blood upon BBB weakening (Zipser et al., 2007; Henriksen et al., 2014). Even if the lesion occurred in the brain, the blood biomarker also could represent the change in AD (Thambisetty and Lovestone, 2010). Additionally, the smaller metabolites have a more chance pass through a weakened BBB (Voyle et al., 2016).

Several studies have shown that sphingolipids in plasma may be important biomarkers for AD. Ceramides in plasma have been related to memory loss and reduction of hippocampal volume. Mielke and colleagues showed that plasma levels of the ceramides C22:0 and C24:0 in patients with MCI were lower than those of HCs and AD cases. A higher baseline level of C22:0 and C24:0 could predict memory impairment and volume loss of the right hippocampus for MCI patients 1 year later (Mielke et al., 2010). In that study, longitudinal follow-up revealed that, compared with people with the lowest serum ceramide level, a higher baseline serum ceramide level of C16:0 and C24:0 was associated with a higher risk of AD, whereas the SM level was unchanged. In particular, individuals with the lowest ceramide level did not suffer AD (Mielke et al., 2012). Those findings indicated that blood levels of sphingolipids and ceramides could be related to the extent of cognitive decline, disease severity, and brain atrophy. Han and colleagues undertook a cross-sectional study based on 26 AD cases and 26 HCs. They took serum samples and measured changes in sphingolipids levels. The levels of eight sphingolipids in AD patients were lower than those in HCs. However, the levels of two ceramides (C16:0 and C22:0) were higher in AD patients than those in HCs, and the ratio of ceramides-to-sphingolipids was higher than that in HCs (Han et al., 2011). It’s indicated that the disorder levels and constituents of sphingolipids in CSF could be reflected in the blood (Mielke et al., 2010). In addition, the levels of sphingolipids and ceramides changed with aging.

Changes in levels of phospholipids have also been investigated. Whiley and colleagues used MA based on liquid chromatography–mass spectrometry and nuclear magnetic resonance on serum samples from 70 individuals. They found that in AD, levels of three PCs (PC 16:0/20:5, PC 16:0/22:6, and PC 18:0/22:6) were lower than those in HCs, and could be diminished in AD cases (Whiley et al., 2014). That finding was consistent with a subsequent validation study involving a larger cohort (*n* = 141). After conducting analyses of receiver operating characteristic (ROC) curves, the three PCs, combined with ApoE status, gave an area under the ROC curve of 0.828. The authors did not mention changes in PCs with similar side chains or changes in the choline metabolic pathway, which suggested that a specific type of lipid was destroyed in AD patients. Some scholars have postulated that the same PCs are related to poor memory performance in older individuals without dementia and that phospholipid metabolism is a common factor in AD and age-related cognitive decline (Simpson et al., 2016). Klavins and collaborators reported lower concentrations of 10 phospholipids (PC aa C36:6, PC aa C38:0, PC aa C38:6, PC aa C40:1, PC aa C40:2, PC aa C40:6, PC ae C40:6, lyso PC aa C18:2, propionyl acylcarnitine (C3), and C16:1-OH) in AD patients, MCI cases, and HCs over a 5-year period (Li et al., 2016). They reported a group of lipid metabolites that could be used to differentiate MCI patients or AD cases from HCs within 2–3 years. Similarly, a lower concentration of PC aa C36:6 in MCI and dementia and the ratio of PC aa 34:4/lyso PC C18:2 could clearly distinguish AD cases or MCI patients from HCs with an accuracy of 82–85% (Klavins et al., 2015). Casanova and colleagues could not replicate those findings using a larger cohort with identical methods (Casanova et al., 2016), which emphasizes the importance of reproducibility in different cohorts.

Changes in levels of some low-molecular-weight metabolites (e.g., amino acids and biogenic amines) have been implicated in AD or MCI. Reduction in levels of amino acids with antioxidant properties (Fonteh et al., 2007), changes in concentrations of metabolites associated with mitochondrial function (Maruszak and Żekanowski, 2011), and reduction in the metabolism of medium-chain fatty acids may participate in neurodegenerative hypometabolism, and there may be a supplementary pathway for impaired carbohydrate catabolism or the tricarboxylic acid (TCA) cycle (Ferreira et al., 2010). Also, biomarkers associated with vascular disorders have been detected in the serum of AD patients, such as homocysteine-cysteine disulfide, asymmetric dimethyl-arginine, and phenylalanyl-phenylalanine (Breteler, 2000). Also, disturbed glutamate neurotransmission has been postulated to be a characteristic feature of AD (Lin et al., 2003).

In summary, analyses of the CSF or blood samples of AD patients suggest that amino-acid metabolism, mitochondrial function, neurotransmitter metabolism, and lipid biosynthesis are changed (Trushina et al., 2013). Studies on these low-molecular-weight metabolites (Table 2) may provide valuable biomarkers for AD diagnosis.

## Analyses of the Metabolic Pathways Associated With Alzheimer’s Disease

**FIGURE 4:**
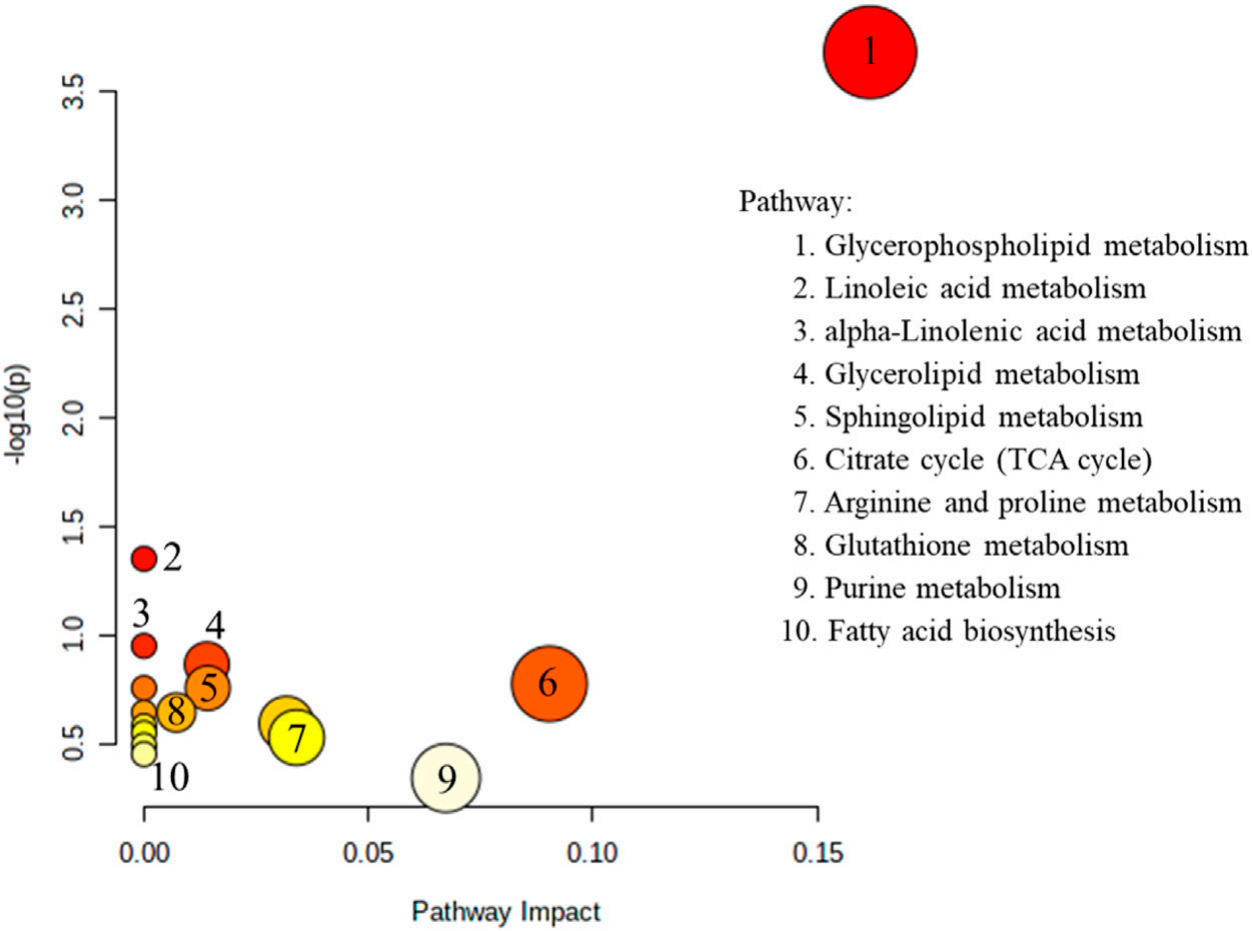
The metabolites reported in the brain and blood (Tables 2, 3, excepted small molecular amino acid) view analyzed by MetaboAnalyst 5.0.

Pathway analyses based on brain and blood samples using MetaboAnalyst 5.0 are shown in Figure 4. Ten metabolic pathways were disordered: glycerophospholipid metabolism; metabolism of linoleic acid; metabolism of alpha-Linolenic acid; sphingolipid metabolism; glycerolipid metabolism; citrate cycle; arginine and proline metabolism; fatty-acid biosynthesis and glutathione metabolism; and purine metabolism. Pathway analyses suggest that lipid metabolism and amino acid metabolism are changed significantly, but purine metabolism is altered only slightly, in AD patients. Recently, studies based on whole-transcript expression on brain tissue indicated stage- and region-dependent deregulation of purine metabolism in AD (González-Domínguez et al., 2014). In addition to intracellular signaling, purines and their products can function as extracellular signals between neurons or neurons and glial cells equipped with appropriate receptors (Ipata et al., 2011). Adenosine (an important neuroprotective factor) is responsible for regulating, integrating, and “fine tuning” neuronal activities and influencing relevant brain functions (sleep and arousal, cognition and memory, and neuronal damage and degeneration) by acting as an extracellular molecule *via* specific adenosine receptors (Anisur, 2009).

**TABLE 3:** Metabolic biomarker in brain or CSF for Alzheimer’s disease patients in clinical.

Metabolites	Subject	Population (sample size)	Sample type	Platform	Implication	Study
Acylcarnitine, propionylcarnitine, lysoPCa C17:0, lysoPCa C18:0, PC aa C38:4, PC aa C40:4, PC aa C40:5, PC aa C40:6, PC ae C34:0, PC ae C34:2, PC ae C36:0, PC ae C36:3, PC ae C36:4, PC ae C40:1, PC ae C42:3, serotonin, spermidine, sphingolipids, SM C16:0, SM C16:1, SM C18:1, SM C24:1, SM C26:1, SM (OH) C14:1, SM (OH) C22:1, SM (OH) C22:2, SM (OH) C24:1	AD, CON	Brain: AD = 15; CON = 14; ASYMAD = 15 (asymptomatic Alzheimer's disease, ASYMAD) Blood:(Prodromal AD) CON = 216; MCI = 366; AD = 185	Brain and Blood	Biocrates AbsoluteIDQ-p180	Sphingolipids and glycerophospholipids could discriminate AD and CN samples with an accuracy 83.33%; SM C16:0, SM C18:1, SM C16:1, and hydroxysphingomyelin (SM (OH) C14:1) were consistently associated with the severity of AD pathology. High concentrations of all four sphingolipids (SM C16:0, SM C16:1, SM C14:1, SM C18:1) increased the risk of conversion to incident AD in the future	Varma et al. (2018)
		(Preclinical AD) N = 92 converters;				
		*N* = 15 non-converters				
Decanoylcarnitine [C10], pimelylcarnitine [C7-DC], tetradecadienylcarnitine [C14:2], PC aa C30:0, PC ae C34:0, PC ae C36:1	AD, MCI, CON	Brain: CON = 51; MCI = 32; AD = 28	Brain and Blood	Biocrates AbsoluteIDQ-p180	Higher levels of acylcarnitines decanoylcarnitine (C10), pimelylcarnitine (C7-DC), and tetradecadienylcarnitine (C14:2) could significantly predict incident of AD, independent of age, sex, and education. (C14:2) was associated with AD.	Huo et al. (2020)
		Blood: CON = 433; MCI = 97; AD = 85				
Norepinephrine, 3-methoxytyramine, alpha-tocopherol, 5-hydroxytryptophan, methoxy-hydroxyphenyl glycol, ascorbate, tyramine, guanosine, vanillylmandelic acid, serotonin, glutathione, hypoxanthine, L-dopa, 3-hydroxyanthranilic acid, methionine, 2-hydroxyphenylacetic acid, xanthine, homovanillic acid, 4-hydroxyphenyllactic acid, xanthosine, tryptophan, N-acetylserotonin, uric acid, tyrosine, kynurenine, 5-hydroxyindoleacetic acid, 2,4-dihydroxyphenylacetic acid, cystine, indoleacetic acid	AD, MCI, CON	AD = 15; CON = 15	CSF (Postmortem)	LC-ECA	Alterations in tyrosine, tryptophan, purine, and tocopherol pathways in AD; reductions in norepinephrine and its related metabolites	Kaddurah-Daouk et al. (2011)
Glutamine, piperine, m/z 246.9550	AD, MCI, CON	AD = 93; MCI = 45; CON = 59	Plasma and CSF	LC–MS	AD was associated with elevated levels of glutamine and a halogen-containing compound and reduced levels of piperine	Niedzwiecki et al. (2020)
C12, C14:1, C16:1, C18, PC ae C36:2, PC ae C40:3, PC ae C42:4, PC ae C44:4, SM (OH) C14:1, SM C16:0, SM C20:2, a-AAA, valine	AD, MCI, CON	AD = 175; MCI = 356; CON = 199	Plasma and CSF	AbsoluteIDQ-p180 and UPLC-TQ-S MS	Sphingomyelins and ether-containing phosphatidylcholines were altered in preclinical biomarker-defined AD stages	Toledo et al. (2017)
Uracil, xanthine, uridine, dopamine–quinone, caproic acid, vanylglycol, histidine, pipecolic acid, creatinine, taurine, sphingosine-1-phosphate, tryptophan, and 5 -methylthioadenosine	AD, MCI, CON	AD = 21; AMCI = 33; CON = 21	CSF	UPLC-Q-TOF-MS	A group of potential biomarkers in CSF samples, Accuracy of 98.7%	Ibáñez et al. (2013)
Acetate, glutamate, succinate, glutamine, Aspartate, creatine, ethanolamine, choline, carnitine, taurine, glycine, isoleucine, serine, myoinositol, N-acetylaspartate, inosine, tyrosine, phenylalanine, hypoxanthine, nicotinate	AD, CON	AD = 15; CON = 15	Brain	1H-NMR	The paired metabolites ratios (alanine/carnitine) were more powerful discriminating tools, AUC = 0.76	Graham et al. (2014)
dGMP, glycine, xanthosine, inosine diphosphate, guanine, deoxyguanosine	AD, CON	AD = 57; CON = 34	Brain	LC-MS	Deregulation of purine metabolism in AD	Ansoleaga et al. (2015)

**FIGURE 5:**
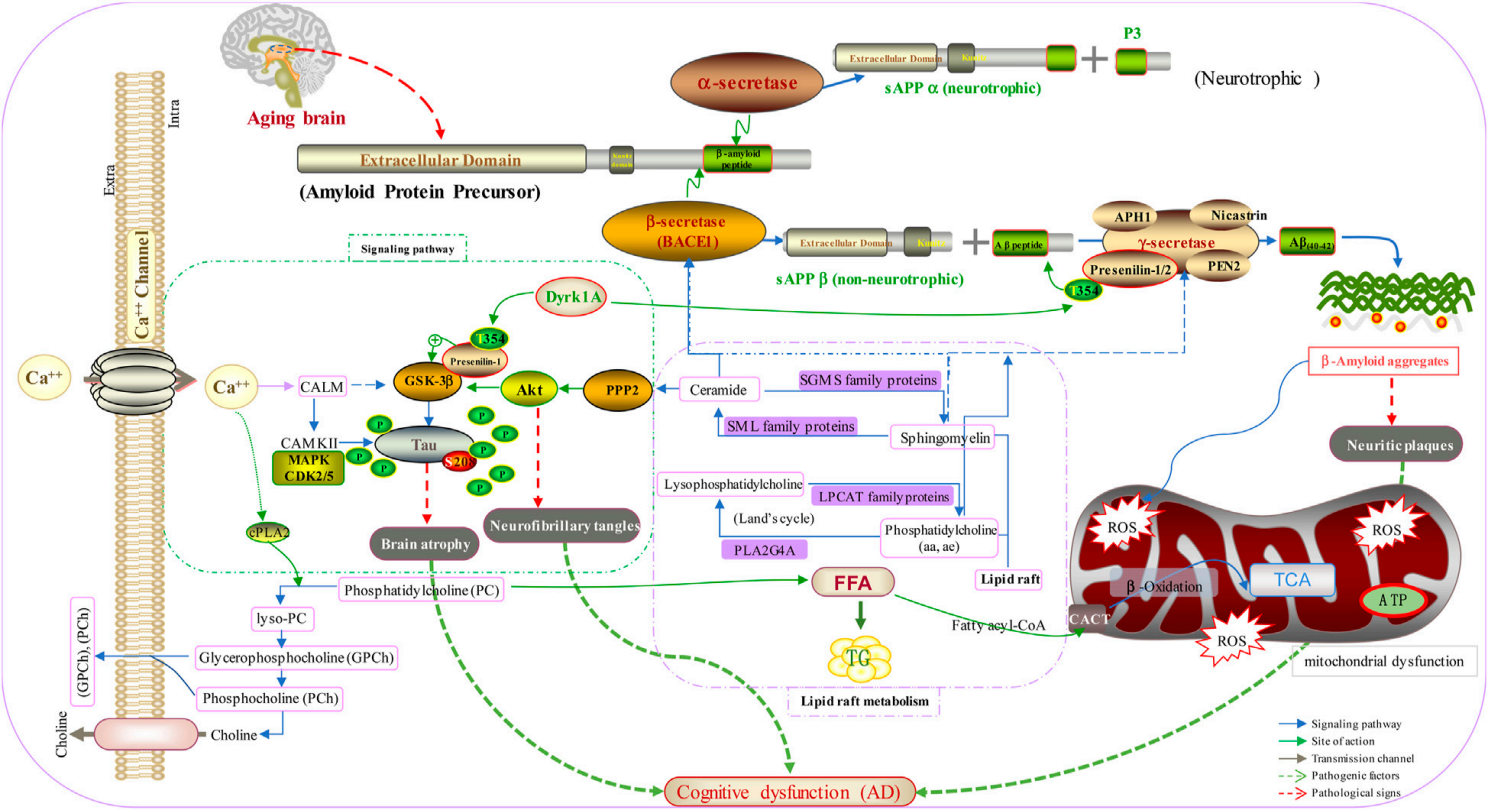
Mechanism based on the brain biomarkers for AD.

A mechanism based on brain biomarkers for AD is shown in Figure 5. In the normal brain, APP is hydrolyzed by α-secretase produced by neurotrophic metabolism to produce sAPPα and P3. However, with aging of the brain, activation of β-secretase and γ-secretase produces Aβ aggregates, which results in neuritic plaques and mitochondrial dysfunction. In addition, disordered metabolism of lipid rafts and Ca^2+^ imbalance accelerate the phosphorylation of tau protein, and β-secretase is activated through glycogen synthase kinase 3 beta and protein kinase B. These actions result in brain atrophy, neurofibrillary tangles, and, ultimately, AD.

## Future Prospects

Current diagnostic methods for AD are slow, expensive, and require several clinical disciplines. The first step is a neuropsychological test, which is suitable only for symptomatic patients. These diagnostic approaches are not suitable in early-stage AD because they only detect if brain function has been damaged irreversibly (Contino et al., 2013). Moreover, AD treatment aims to relieve symptoms by improving neurotransmission and involves use of cholinesterase inhibitors and antagonists of N-Methyl-D-aspartic acid receptors. However, these drugs treat symptoms and do not prevent AD progression. The preclinical stage of AD provides an opportunity to reduce the risk of developing AD. Earlier diagnosis using CSF samples (invasive, expensive, and risk of sequelae) and brain imaging (expensive and limited availability) is difficult in most clinical settings. Nevertheless, biomarkers in CSF samples are used commonly in clinical research. Although using biomarkers in CSF and brain imaging are helpful, a primary screening tool is needed for AD because of the aging societies found worldwide. Therefore, patients who could benefit from these strategies must be identified and treated in a timely manner. Blood-based biomarkers for early screening would be very advantageous because sample collection is easy and cost-effective. MA could have huge potential in AD-biomarker investigation and the early diagnosis of AD. A blood sample has also been increasingly explored for AD biomarker over the last 10 years, due to a reliable panel of biomarker given much hope. The biomarker researches should conduct a longitudinal investigation of subjects, who subsequently then convert to clinical AD. Lipids and amino acids could be candidate biomarkers for the early diagnosis of AD.

The inconsistent results obtained from different studies are a major limitation in the development of AD biomarkers. This problem arises due to a lack of standardized protocols in terms of the collection, storage, preparation of samples, and analytical methodologies (Arnerić et al., 2017). In addition, heterogeneity of participants among different trials is another challenge, which is usually due to differences in age, sex, demographic characteristics, neuropathology, and the different underlying genetics in each individual. A standardized guideline for preanalytical processing of samples and validation of potential biomarkers are needed to overcome these problems. Finally, a clear definition of the preclinical phenotype is necessary for research on modifying the course of AD.
